# Whole-exome sequencing in children with dyslexia implicates rare variants in *CLDN3* and ion channel genes

**DOI:** 10.1007/s00439-025-02796-0

**Published:** 2025-12-24

**Authors:** Krzysztof Marianski, Joel B. Talcott, John Stein, Anthony P. Monaco, Simon E. Fisher, Dorothy V.M. Bishop, Dianne F. Newbury, Silvia Paracchini

**Affiliations:** 1 School of Medicine, University of St Andrews, St Andrews, UK; 2 Institute of Health and Neurodevelopment, College of Health and Life Sciences, Aston University, Birmingham, UK; 3 Department of Physiology, University of Oxford, Oxford, UK; 4 Office of the President emeritus, Tufts University, Medford, MA USA; 5 Language and Genetics Department, Max Planck Institute for Psycholinguistics, Nijmegen, Netherlands; 6 Donders Institute for Brain, Cognition and Behaviour, Radboud University, Nijmegen, Netherlands; 7 Department of Experimental Psychology, University of Oxford, Oxford, UK; 8 Department of Medical and Biological Sciences, Oxford Brookes University, Oxford, UK; 9 Biomedical Research Centre, Centre for Human Genetics, Nuffield Dept Medicine, University of Oxford, Oxford, UK

## Abstract

Dyslexia is a specific difficulty in learning to read that affects 5–10% of school-aged children and is strongly influenced by genetic factors. While previous studies have identified common genetic variants associated with dyslexia, the role of rare variants has only recently begun to emerge from pedigree studies and has yet to be systematically tested in larger cohorts. Here, we present a whole-exome sequencing (WES) study of 53 individuals with dyslexia, followed by an analysis in 38 cases with reading difficulties and 82 controls assessed with reading measures. Of the 22 genes that had high-impact variants filtered through stringent bioinformatic approaches in at least three dyslexia cases, five genes were validated in the follow-up analysis: *CACNA1D*, *CACNA1G*, *CLDN3*, *CNGB1*, and *CP*. Notably, a specific variant (7-73769649-G-A; c.C401T; p.P134L) in the *CLDN3* gene was identified in six independent cases, showing a four-fold higher frequency compared to population reference datasets. *CACNA1D* and *CACNA1G* encode subunits of voltage-gated calcium channels expressed in neurons, and variants in both genes have been implicated in neurodevelopmental disorders such as autism spectrum disorder (ASD) and epilepsy. Segregation analyses in available family members were consistent with patterns of dominant inheritance with variable expressivity. In total, high-impact variants in the five genes of interest were found in 26% (*N* = 14) of individuals of the discovery cohort. Overall, our findings support the involvement of rare variants in developmental dyslexia and indicate that larger WES studies may uncover additional associated genes.

**Supplementary Information:**

The online version contains supplementary material available at 10.1007/s00439-025-02796-0.

## Introduction

Dyslexia is a neurodevelopmental condition characterised by a specific difficulty in learning to read, occurring in the absence of other causes such as sensory or neurological problems or lack of educational opportunity (Erbeli et al. 2021). Regardless of culture and spoken-language, dyslexia affects 5%–10% of children, with a male: female ratio ranging from about 3:1 to 5:1 (Arnett et al. 2017), and often co-occurs with other neurodevelopmental conditions such as developmental language disorders (DLD) and attention deficit hyperactivity disorder (ADHD) (Snowling et al. 2020; Daucourt et al. 2020). Dyslexia can be considered as the lower end of a continuum of reading abilities, which follows a normal distribution in the general population (Shaywitz et al. 1992). Twin studies have shown a strong genetic component with heritability estimates of 40–70% both for dyslexia and reading abilities (DeFries and Alarcon 1996; Hensler et al. 2010; Andreola et al. 2020). Moreover, having a first degree relative with dyslexia is the most consistently identified risk factor (Snowling and Melby-Lervåg 2016). These observations have motivated molecular studies, but the identification of specific genes has, until recently, been hindered by difficulties in assembling sufficiently powered sample sizes. Lack of national screening programs and heterogeneous assessment and diagnostic criteria pose challenges in recruiting study participants and combining them across studies (Erbeli et al. 2021). Initial molecular studies highlighted a handful of genes, including *ROBO1*,* DCDC2*,* KIAA0319* and *DYX1C1* (Paracchini et al. 2007, 2016; Newbury et al. 2014). However, associations with these genes did not consistently replicate (Erbeli et al. 2021).

A breakthrough was made possible by a large (51,800 cases and 1,087,070 controls) genome-wide association study (GWAS) conducted with self-reported dyslexia diagnoses by customers of the direct-to-consumer company 23andMe (Doust et al. 2022). This study identified 42 independently statistically significant associated loci. About half of these had previously been associated with cognitive phenotypes, while the other half suggested effects more specific to dyslexia. A gene-based analysis reported associations with 173 genes. This study also facilitated the generation of dyslexia polygenic scores, which showed significant associations with the reading-related measures available for some cohorts of the GenLang consortium (https://www.genlang.org/), an international project aimed at dissecting the genetics of language-related traits, including reading abilities. An independent quantitative meta-GWAS of reading- and language-related measures in the GenLang cohorts (*N* ~ 34,000) identified only a single genome-wide significant locus but highlighted significant genetic correlations across language-related cognitive domains and traits derived from neuroimaging data (Eising et al. 2022). The SNP-based heritability from these GWAS efforts ranged between 0.13 and 0.26. Although larger GWAS are expected to identify additional common variants associated with dyslexia, it is likely that rare variants, e.g. those with a minor allele frequency (MAF) < 1%, will also play a role.

Whole-exome (WES) and whole-genome sequencing (WGS) studies have demonstrated the role of rare variants in most complex traits (Momozawa and Mizukami 2021) including for neurodevelopmental conditions like autism spectrum disorder (ASD) (Wang et al. 2022), schizophrenia (Howrigan et al. 2020), bipolar disorder (Forstner et al. 2020) and childhood apraxia of speech (CAS) (Eising et al. 2019; Hildebrand et al. 2020; Kaspi et al. 2023). Rare variants have been reported also for conditions that frequently co-occur with dyslexia, such as ADHD (Rajagopal et al. 2022) and language impairment (Villanueva et al. 2015; Chen et al. 2017; Devanna et al. 2018; Martinelli et al. 2021). Rare variant analyses and WES screenings focussing specifically on dyslexia are limited. *DYX1C1*, the first risk gene reported as a dyslexia candidate was found to be disrupted by a translocation in one individual (Taipale et al. 2003) and several loci were suggested by linkage studies in family cohorts (Scerri and Schulte-Korne 2010; Rubenstein et al. 2014). Segregation analyses in large pedigrees suggested the role of variants in the *CEP63* (Einarsdottir et al. 2015), *NCAN* (Einarsdottir et al. 2017), and *SEMA3C* (Carrion-Castillo et al. 2021) genes. Large population-based cohorts like the UK Biobank, for which WES data have been generated at scale, are not well-suited for genetic studies of dyslexia because data on dyslexia diagnosis are not available or reliable and reading-related measures have not been collected (Erbeli et al. 2021). Furthermore, it has been shown that rare variants detected in clinical cohorts might not show signals in population samples because of incomplete penetrance *(i.e.* when not all individuals with the variant exhibit the trait) or variable expressivity (when different degrees of a phenotype are associated with the variant) (Kingdom and Wright 2022). A possible explanation is that the effects of rare variants are enhanced in clinical cohorts because of a higher burden of risk factors.

Given the increasing evidence supporting the role of rare variants in a wide range of conditions including neurodevelopmental traits, we report a proof-of-principle WES study for dyslexia. We analysed 53 unrelated individuals with dyslexia. An independent cohort of 38 cases with reading difficulties (RD) and 82 typically developing (TD) controls, assessed with the same quantitative test as part of a twin study on language-related problems and co-occurring conditions was analysed to follow up the initial findings. Our findings highlight rare variants in several genes.

## Materials and methods

### Discovery cohort

We analysed a discovery sample of 53 unrelated cases selected from two existing cohorts recruited for genetic studies of dyslexia and which have been previously described (Table 1) (Scerri et al. 2017). The first cohort includes 290 families (689 siblings, 580 parents). Initially the families were ascertained if the proband had a single-word reading (SWR) score > 2 standard deviations (SDs) below that predicted by their performance IQ and at least one other sibling with a record of reading difficulties. These criteria identified some probands with high IQ scores and reading scores within the normal range and therefore the final third of families was recruited with revised criteria: the SWR score had to be ≥ 1 SD below the population mean in combination with a performance IQ ≥ 90 and the requirement of reading difficulty in another sibling was dropped.

The second cohort comprises 592 singletons with dyslexia or reading problems. The cases entered the study if they had a SWR score ≤ 100 (at chronological age) and > 1.5 SDs below that predicted by their IQ scores.

Both cohorts were recruited in the South of England at Dyslexia Research Centre clinics in Oxford and Reading or the Aston Dyslexia and Development Unit in Birmingham. All children (probands and their siblings) were individually assessed with the following core tasks: SWR and single-word spelling accuracy (SPELL)(Elliot 1983), single non-word reading (NWR) (Castles and Coltheart 1993), phonological awareness (PA) (Frederickson 1997), irregular word reading (IWR) (Castles and Coltheart 1993), orthographic coding (ORTH) (Olson 1994), as well as measures of verbal (VIQ) and performance (PIQ) IQ (Wechsler et al. 1992) (See Supplementary Tables 1 and Supplementary Figure S1 for more details on these measures). Parents in the family cohort were not formally assessed but some of them provided information about their own and their family’s history of dyslexia or reading problems by questionnaire during an interview. A formal diagnosis of language impairment, autism or ADHD was an exclusion criterion at the time of recruitment for both cohorts.

For the current study, individual cases were selected from these two cohorts if they scored 1SD below the mean of at least one reading measure and had a performance IQ above or equal to −1SD, and then prioritised for having a high-quality DNA sample available. These criteria allowed us to select individuals with similar characteristics avoiding the inclusion of individuals with high IQ scores and reading performance in the normal range who entered the study in the initial phase of recruitment for the dyslexia family cohort.

In total, 53 unrelated cases were selected: 33 from the family cohort and 20 from the singleton cohort. WES data were also generated for the available family members (*N* = 14) of carriers of variants in the top five genes identified in this study for segregation analysis.

### Independent cohorts for follow-up analyses

We follow-up the top findings in independent cohorts derived from a UK twin cohort (*N* = 194 twin pairs) recruited to study language or literacy problems (Wilson and Bishop 2018). The assessment of this cohort with reading-related measures allowed us to identify a set of cases by applying the same criteria used in the discovery cohort as wells as a set of typically developing (TD) controls. We refer to the cases from this cohort as having reading difficulties (RD), as opposed to dyslexia, because they did not receive a formal diagnosis. RD cases and TD controls were selected based on the following criteria. For consistency with the inclusion criteria applied to the discovery cohort, the RD cases were required to have a PIQ above or equal to −1 SD and at least one score more than 1 SD below the expected level on any of the following tests: single word reading (SWR), non-word reading (NWR) (Torgesen et al. 1999), text reading accuracy (ACC), comprehension (COMP) or reading rate (RR) from the NARA-II (Neale 1999). See Supplementary Tables 1 and Supplementary Figure S1 for more details on the tests.

TD controls were selected based on a PIQ of no more than 1 SD below the expected level for their age and a SWR score above the mean. This cut-off was chosen to ensure that the controls would not have any reading problems and is the same as used in previous studies (Scerri et al. 2011). Children with a Development and Well-Being Assessment (DAWBA) diagnosis of ASD (Goodman et al. 2000) were removed from both groups.

If both twins in a pair met the criteria for inclusion, one twin was randomly selected for the study. This process resulted in 38 RD cases and 82 TD controls with good reading scores. Descriptive statistics and mean phenotypic scores for these two groups are shown in Table 1. WES and phenotypic data were available for their twin siblings for follow up analyses.

Siblings and family members of the dyslexia and RD cases were assigned to a category of mild reading difficulties if they scored below 1 SD on at least one reading measure or had an average score across all reading measures at least 0.25 SD below the mean.

### Bioinformatics analysis

WES data for all samples were generated using Illumina technology (NovaSeq 6000, Q30 ⩾ 80%, with 50X coverage). Raw sequences were trimmed using Trimmomatic (Bolger et al. 2014). Trimmed reads were mapped to the human reference genome (GRCh38) using bwa-mem (https://github.com/lh3/bwa). Picard tools (http://broadinstitute.github.io/picard/) were used for read-groups replacement, removal of PCR duplicates, and base recalibration. The pre-processed reads were indexed using SAMtools (https://www.htslib.org/) and called with DeepVariant v1.4.0 (Poplin et al. 2018). VCF files were annotated with ANNOVAR (Wang et al. 2010) to identify high-impact variants.

In accordance with the ACMG guidelines (Richards et al. 2015), high-impact variants were defined as rare variants (PM2) predicted to damage protein function (PP3). Accordingly, variants were removed if they had a quality score < 20, read depth < 10 and a minor allele frequency (MAF) ≥ 1% (based on gnomAD v3.1.2 for Non-Finnish Europeans), or if they were annotated as synonymous, intronic or intergenic variants. To be retained, variants had to be predicted as damaging by all five predictive algorithms (SIFT, PolyPhen2, LRT, MutationTaster and FATHMM) or already annotated as pathogenic or likely pathogenic in ClinVar for any disorders (https://www.ncbi.nlm.nih.gov/clinvar/). The same filtering criteria were applied to the discovery, RD and TD cohorts.

Genes were selected as candidates if high-impact variants, as defined above, were detected in three or more independent cases in the discovery cohort in accordance with Genomics England guidelines which prioritises genes using a “traffic-light” system to estimate the level of confidence for involvement of a gene in a disorder without implementing formal statistical tests (https://panelapp.genomicsengland.co.uk/) (Martin et al. 2019). This threshold aligns with the ACMG guidance (See Note 2 in Table 3 in Richards et al. 2015) which emphasises the importance of recurrence of variants in the same gene across unrelated individuals for prioritisation, when case-control studies are not large enough to reach statistical significance. In line with this recommendation, to warrant further follow-up, the gene also had to carry at least one high-impact variant in the RD cases and no such variants in the TD controls. The selected variants were manually inspected with the Integrated Genome Viewer (IGV; (Robinson et al. 2011). Statistical significance for case-control comparisons was assessed using Fisher’s exact test.

Genomic localisation of the variants of interest was based on UniProt and InterPro data. Exonic locations were accessed from UCSC Table Browser hg38.refGene. All analysis was conducted on the UK’s Crop Diversity Bioinformatics HPC (Percival-Alwyn et al. 2024).

### Post-WES analyses

The overlap between the genes identified as candidates in the discovery cohort and those found to be associated in the dyslexia GWAS by Doust et al. (2022) was tested with the GeneOverlap package in R (https://rdrr.io/bioc/GeneOverlap/).

Gene-set enrichment was performed in FUMA (GENE2FUNC tool https://fuma.ctglab.nl/) (Watanabe et al. 2017) using Gene Ontology classifications and expression data from GTExv8.0 which includes gene expression information across 54 adult tissues, including 13 brain regions (Lonsdale et al. 2013), and BrainSpan which includes averaged brain gene expression data across 29 different ages, from 8 weeks post-conception to 40 years (Kang et al. 2011). Between them, these two datasets allow both a spatial and temporal investigation of brain gene expression. Genes identified in the WES analyses were compared against all protein-coding genes, with Benjamini-Hochberg (false discovery rate, FDR) correction applied for multiple testing.

Brain expression levels for top genes were extracted from GTEXv8.0 (Lonsdale et al. 2013) within FUMA and reported as the maximum observed normalised gene expression across 13 brain regions: amygdala, anterior cingulate cortex, basal ganglia, cerebellar hemisphere, cerebellum, cortex, frontal cortex, hippocampus, hypothalamus, nucleus accumbens, putamen, spinal cord, and substantia nigra. Expression in single cells was evaluated in 81 cell types and across single brain nuclei (Siletti et al. 2023) by visualising single-cell and single nuclei RNA sequencing data through the Human Protein Atlas https://www.proteinatlas.org/.

## Results

WES analysis in the discovery cohort (*N* = 53) identified 580 high-impact variants (as defined in Methods) across 512 genes (Supplementary Table 2). On average, each individual carried 11 high-impact variants.

To investigate overlaps with previous findings from analyses of common variants, we compared the identified genes with the 173 genes reported in the gene-based analysis of the dyslexia GWAS by Doust and colleagues (Doust et al. 2022). There was no statistically significant overlap (*P* = 0.16) and only seven of the GWAS-identified genes carried high-impact variants in our current study: *CUX2*,* HSPB2*,* INA*,* MITF*,* SCN5A*,* SGCD*, and *WDR38*. Among these, *INA* was the only gene that carried high-impact variants in more than one independent case (*N* = 2; Supplementary Table 2).

**Table 1:** Descriptive statistics of the cohorts

Cohort	*N* (M)	Age	PIQ	SWR	NWR	SPELL	IWR	ORTH	PA	AVG
Discovery	53 (40)	12	0.17	−2.04	−1.52	−2.30	−2.39	−1.79	−1.25	−2.01
Follow-up			**PIQ**	**SWR**	**NWR**	**ACC**	**COMP**	**RR**		**AVG**
RD cases	38 (22)	8	−0.10	−0.71	−0.80	−1.05	−1.00	−0.67		−0.85
TD controls	82 (36)	8	0.47	0.97	0.73	0.34	0.27	0.62		0.60
T-stat			3.77	11.21	10.23	11.23	10.12	8.22		13.46
*p*			1.55E-3	1.61E-19	3.50E-17	2.02E-19	8.84E-17	2.09E-12		1.37E-25

Phenotypic scores are represented as mean z-scores, which have been standardized based on population distributions with mean = 100 and SD = 15. *N*: sample size; *M*: male; *Age*: mean age at assessment in years; *PIQ*: performance IQ, *SWR*: single word reading;*NWR*: non-word reading; *SPELL*: single word spelling accuracy;*IWR*: irregular word reading; *ORTH*: orthographic coding; *PA*: phonemic awareness; *AVG*: average of reading measures; *ACC*: NARA-II accuracy; *COMP*: NARA-II comprehension; *RR*: NARA-II reading rate; *T-stat*: absolute t-test statistic; *p*:*p-value*: Bonferroni adjusted for six tests.

The 512 genes identified in our analysis did not include any previously reported candidate genes associated with dyslexia through common (*i.e. KIAA0319* and *DCDC2)* or rare (*i.e. CEP63*,* DYX1C1*,* NCAN*,* ROBO1*, and *SEMA3C*) variants. However, our annotations for disease associations highlighted two different variants in *ZGRF1*, a gene previously implicated in CAS (Peter et al. 2016), in two independent cases. Gene pathway enrichment analysis showed that the top associated pathways were action potential for the biological processes (BP, *p*_adj_ = 3.55E-6) category, membrane protein complex for the cellular component (CC, *p*_adj_ = 5.23E-14) category, and adenyl nucleotide binding for the molecular function (MF, *p*_adj_ = 4.56E-14) category (Supplementary Tables 3–5).

Analyses of gene expression profiling data did not show enrichment for brain expression in any specific regions when testing GTEx v8 general tissue types (Supplementary Table 6) or BrainSpan datasets (Supplementary Tables 7 and 8).

There were 22 genes that presented high-impact variants in at least three independent cases (Supplementary Table 9). The gene with the highest number of high-impact variants was *CFTR*, with six different variants identified in seven individuals. This was followed by *CLDN3* which had the same 7–73769649-G-A; c.C401T; p.P134L variant reported in five unrelated individuals. The top associations in gene pathways enrichment analysis for these genes were membrane depolarization (BP, *p*_adj_ = 0.01; Supplementary Table 10), transporter complex (CC, *p*_adj_ = 4.26E-3; Supplementary Table 11), and high voltage-gated calcium channel activity (MF, *p*_adj_ = 2.63E-3; Supplementary Table 12). This group of genes did not exhibit specific patterns of brain expression (Supplementary Tables 13–15).

**Fig. 1:**
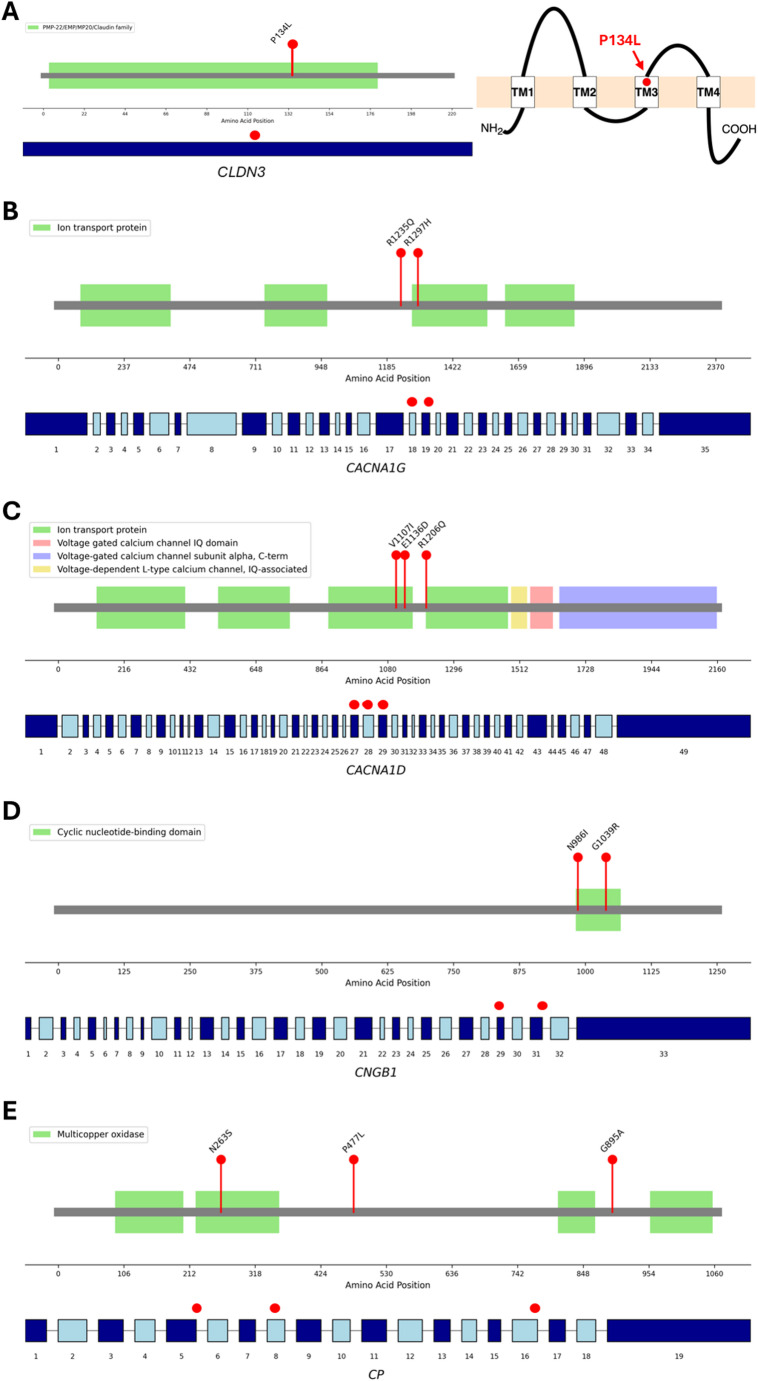
Schematic showing high-impact variants in five top genes. The location of high-impact variants (red dots) is shown in relation to exons (lower tracks) and protein domains (upper track) for the (A) *CLDN3*, (B) *CACNA1G*, (C) *CACNA1D*, (D) *CNGB1*, and (E) *CP* genes. A) A 2D structure of the single-exon CLDN3 gene shows that the 7–73769649-G-A; c.C401T; p.P134L variant leads to the P134L amino acid change in the third transmembrane (TM3) domain of the protein

We then followed-up the 22 genes in 38 individuals with reading difficulties (RD cases) and in 82 typically developing (TD) controls (Table 1). To warrant further follow-up a gene required at least one high-impact variant to be present in the RD cases, even if this involved a different variant from that identified in the discovery cohort. Additionally, no variants identified in the discovery and RD cases could be present in the TD group. This pipeline resulted in five genes: *CLDN3*, *CACNA1G*, *CACNA1D*, *CNGB1*, and *CP*. An additional 7–73769649-G-A rare allele in *CLDN3* was identified in the RD cases (Table 2). For three genes (*CACNA1G*, *CACNA1D*, and *CNGB1)*, high-impact variants tended to cluster in adjacent exons and, consequently, in spatially close regions of the resulting protein (Fig. 1).

Gene expression profiles derived from public repositories showed that three genes of the five genes (*CLDN3*,* CACNA1G*, and *CACNA1D*) had the highest expression in the cerebellum. Single cells transcriptomic data showed specific expression in neuronal cells for *CACNA1G*, *CACNA1D* and *CNGB1* (Supplementary Figure S2). *CP* showed high expression in different cell types including glial cells. *CLDN3* did not show high expression in neuronal cell, but its expression was detected in cone and rod photoreceptor cells. All five genes were expressed in these two cell types. Evaluation of clusters of cell types specific to 11 brain nuclei revealed wide-spread expression for *CLDN3*,* CACNA1G*, and *CACNA1D*, while *CLDN3* and *CP* showed a similar pattern of expression specific to choroid plexus epithelial and ependymal cells although the expression level for *CLDN3* was lower (Supplementary Figure S3).

All high-impact variants occurred in a heterozygous state. One individual carried high-impact variants in three of these genes (*CNGB1*,* CLDN3* and *CACNA1D)* and two individuals carried high-impact variants in two genes (*CACNA1D* and *CLDN3; CACNA1G* and *CLDN3).*

We compared reading scores between the carriers and non-carriers of the *CLDN3* variant which showed highest recurrence in the discovery cohort (Supplementary Table 16). On average the carriers of this variant exhibited lower scores on all six reading-related measures. The RD case who carried the 7–73769649-G-A rare allele exhibited a high PIQ (z-score = 1.13), and extremely low reading scores (i.e. SWR z-score = −3; average reading z-scores = −2.23). Their dizygotic twin sibling shared the same variant and did not meet criteria for assignment to either the RD or the TD group. This sibling presented a high PIQ score (z-score = 1.6) and all five reading scores were below the mean (average z-score = −0.68), meeting criteria for mild reading problems.

Except for the *CLDN3* variant, which was the only one present in at least five cases, formal case-control comparisons were not possible given the small sample size and rarity of the variants in the other genes. The *CLDN3* variant was nominally significantly associated with dyslexia when comparing the discovery and RD cases with either the TD cohort (*P* = 0.03) or the general population (i.e. gnomAD data; *P* = 0.003) (Supplementary Table 17). The small size of our sample needs to be considered when interpreting these results.

Finally, we evaluated the segregation patterns of variants in the five genes, for cases in the discovery cohort where family members were available. Siblings had been assessed using the same battery of tests as the cases. Parents had not been assessed systematically, but for some of them self-reported information for dyslexia diagnoses or reading difficulties were available.

**Fig. 2:**
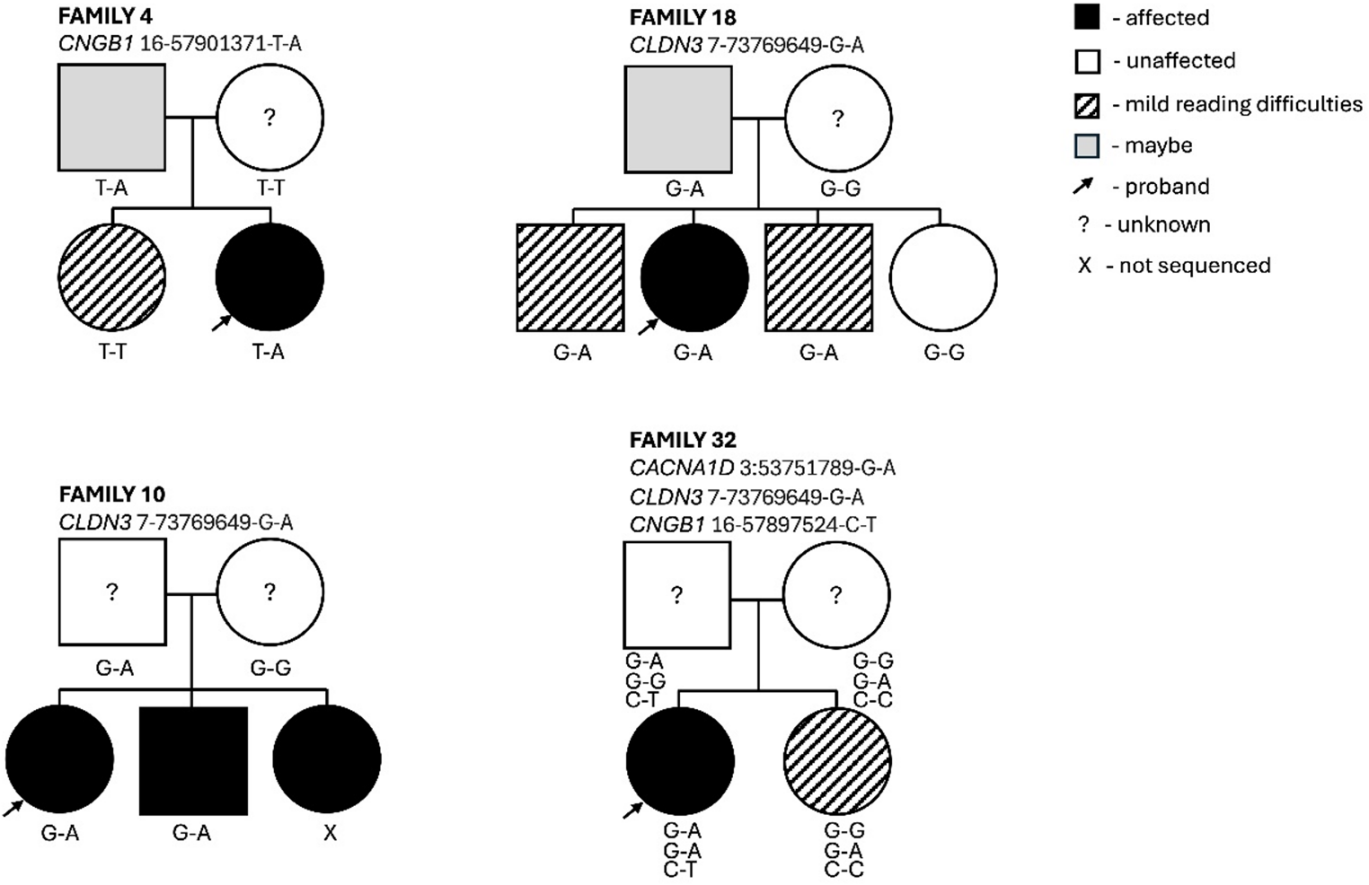
Segregation analysis. Four families were available for segregation analysis of the variants in the five candidate genes. Overall, segregation patterns are consistent with dominant effects with variable expressivity. The phenotypes of the probands and their siblings were defined through a battery of cognitive tests aimed at assessing reading abilities. Parents were not formally assessed, and their phenotypic status was based on self-reported information. Parents who answered “maybe” when asked if they had dyslexia are indicated in grey. A question mark indicates that no information was available for the parents. Mild reading difficulties (striped pattern) in the children were defined as having either at least one of the reading scores lower than 1 SD from the mean or an average score across all reading measures at least 0.25 SD below the mean

No data on family members were available for carriers of variants in the *CACNA1G* and *CP* genes. Only one family was available for *CNGB1* (16–57901371-T-A; c.A2939T; p.N980I), two families were available for *CLDN3* (7–73769649-G-A; c.C401T; p.P134L), and one family had variants in three genes (*CNGB1*: 16–57901371-T-A; c.A2939T; p.N980I; rs201162411, *CLDN3*: 7–73769649-G-A; c.C401T; p.P134L and *CACNA1D*: 3–53751789-G-A; c.G3557A; p.R1186Q; rs745599441 Fig. 2). Overall, the observed segregation patterns were consistent with dominant effects with variable expressivity as all individuals carrying high-impact variants and for whom data were available exhibited some form of reading difficulties.

**Table 2:** Genes meeting our prioritisation criteria

Gene	Variant	gnomAD AF	CADD score	Discovery *N* = 53	RD cases *N* = 38	TD Controls *N* = 82	Max brain Expression**	Nervous system region
CLDN3	7–73769649-G-A; c.C401T; p.P134L; rs139191328	0.0078	33	5	1	0	2.03	Cerebellar Hemisphere
	**All variants**			**5**	**1**	1*		
CACNA1G	17–50600739-G-A; c.G3635A; p.R1212Q; rs150972562	0.0034	28	3	1	0	3.86	Cerebellum
	17–50601149-G-A; c.G3821A; p.R1274H; rs764399144	0.000031	34	1	0	0		
	**All variants**			**4**	**1**	**0**		
CACNA1D	3–53747393-G-A; c.G3259A; p.V1087I; rs768995869	0.0000155	34	1	0	0	1.7	Cerebellum
	3–53749301-G-T; c.G3348T; p.E1116D	.	28.8	1	0	0		
	3–53751789-G-A; c.G3557A; p.R1186Q; rs745599441	0.0000465	34	1	0	0		
	**All variants**			**3**	1*	**0**		
CNGB1	16–57897524-C-T; c.G3097A; p.G1033R; rs148999583	0.0061	34	2	1	0	2.48	Hypothalamus
	16–57901371-T-A; c.A2939T; p.N980I; rs201162411	0.0011	31	1	0	0		
	**All variants**			**3**	2*	**0**		
CP	3–149178609-C-G; c.G2684C; p.G895A; rs139633388	0.0023	27.4	1	1	0	0.87	Spinal cord
	3–149199783-G-A; c.C1430T; p.P477L; rs35331711	0.0042	28.3	1	0	0		
	3–149207611-T-C; c.A788G; p.N263S; rs150303869	0.0006	25.8	1	0	0		
	**All variants**			**3**	**1**	1*		

* variant in the same gene but different from those identified in the discovery cohort

** maximum observed normalised gene expression across 13 brain regions; *RD: R*eading Difficulties; *TD:* Typically Developing; *CADD*: Combined Annotation Dependent Depletion.

## Discussion

We report the results of a WES study of dyslexia conducted in a discovery cohort (*N* = 53) and follow up analyses in 38 RD cases and 82 TD controls, representing the largest analysis of this kind conducted to date. All cases and their siblings were assessed in person with a battery of standardised tests. Our findings highlight five genes with high-impact variants detected in 26% (*N* = 14) individuals of the discovery cohort.

The observation with highest recurrence was a specific rare variant (7–73769649-G-A; c.C401T; p.P134L) in the *CLDN3* gene, which was observed in a total of six unrelated cases across the discovery and RD cohorts (N_total_ = 91). This corresponds to an allele frequency of 3.3%, representing a four-fold increase compared to the frequency of 0.8% reported for this variant in the gnomAD reference dataset. Moreover, this variant was not observed in the TD controls, minimising a potential population structure effect caused by a recruitment bias. The comparisons for this variant yielded nominally significant associations which need to be considered in the context of a small sample that limits the robustness of these findings. The *CLDN3* variant has an allele frequency of less than 1% across all populations reported in gnomAD, except for the Amish population where it is observed at a frequency of 7%. Furthermore, 52 individuals have been identified as homozygous for this variant in gnomAD. However, the absence of systematically collected phenotypic data, particularly for conditions like dyslexia, and the lack of information on the prevalence of dyslexia within the Amish population, prevent drawing further conclusions.

*CLDN3* is a single exon gene spanning 1274 bases in total at 7q11.23, within the critical region deleted in Williams syndrome, a developmental disorder characterized by learning difficulties, distinctive facial features, and cardiovascular problems (Morris and Mervis 2021). This gene encodes Claudin-3, a protein expressed in different epithelia with roles for the formation of tight junctions and the blood-brain-barrier (Günzel and Yu 2013). Depletion of Cldn-3 has been shown to lead to neural tube defects using a chick embryos model system. Single cell transcriptomics data show a specific expression of *CLDN3* in the upper rhombic lip, a transient structure in the developing brain which gives rise to cerebellar neurons, and in choroid plexus cells (Supplementary Figure S3). Single cell transcriptomics in the developing mouse also found that Cldn3 was specifically expressed in motor neurons but only up to E10.5 stage (Delile et al. 2019). These data suggest that *CLDN3* has a tightly regulated role during neurodevelopment. Targeted analysis of *CLDN* genes in 152 patients with spinal neural tube defects (NTDs) identified eleven variants, including a p.A128T missense variant in *CLDN3* (Baumholtz et al. 2020). Overexpression of the *CLDN3* p.A128T variant in chick embryos resulted in a significant increase in NTDs in this model. The 7–73769649-G-A variant identified in this study results in a p.P134L amino acid change, which, like p.A128T, is located in the third transmembrane (TM3) domain of Claudin-3. In both Claudin-3 and Claudin-5, the TM3 domain is critical for the assembly of tight junctions, as demonstrated by studies in HEK293 cells (Rossa et al. 2014). These observations, combined with a high CADD score (= 33; Table 2), predict that the 7–73769649-G-A variant is likely to impact the function of the CLDN3 protein.

Among the five genes were two members of the voltage-gated calcium channel (VGCC) family, *CACNA1D* and *CACNA1G*. VGCC genes are critical for proper brain function, mediating essential calcium-dependent processes such as gene transcription, neurotransmitter release, and neurite outgrowth (Simms and Zamponi 2014). Both *CACNA1D* and *CACNA1G*, which show high level of expression in the cerebellum, have been previously implicated in neurodevelopmental disorders. *CACNA1D* variants have been associated with ASD, global developmental delay and other neurodevelopmental conditions (Rajakulendran and Hanna 2016; Hofer et al. 2020). *CACNA1G* variants have been associated with infantile-onset syndromic cerebellar ataxia (Barresi et al. 2020), severe neurodevelopmental delay, epilepsy (Rajakulendran and Hanna 2016) and intractable seizures (Kunii et al. 2020). Furthermore, we observed high-impact variants in another VGCC gene, *CACNA1C*, in two independent cases from the discovery cohort (Supplementary Table 2). No variants in *CACNA1C* were observed in the TD group.

For the remaining two genes, there is less evidence for a role in neurodevelopment. The *CNGB1* gene encodes the cyclic nucleotide-gated channel beta 1 protein, which is a subunit of the rod photoreceptor cyclic nucleotide-gated channels. These channels ensure a flow of ions into the rod photoreceptor outer segment in response to light-induced changes in intracellular cyclic GMP levels. *CNGB1* variants are associated with retinitis pigmentosa, a degenerative eye disease that affects photoreceptor function (Alshamrani et al. 2020). *CP* encodes ceruloplasmin, a major copper-carrying protein in the blood. Variants in the *CP* gene can lead to aceruloplasminemia, a rare genetic disorder characterised by iron accumulation in the brain and other organs which can result in neurological and systemic symptoms (Yoshida et al. 1995). Elevated levels of iron in the cortical speech motor network have been reported in people who stutter (Cler et al. 2021). Single cell transcriptomic data show high and specific expression in neuronal cells for *CACNA1D*,* CACNA1G* and *CNGB1*, while *CP* shows a pattern similar to *CLDN3* with specific expression in choroid plexus cells (Supplementary Figure S2 and S3). The function of the choroid plexus has been less investigated compared to other brain structures, however recent research has demonstrated its role in neurogenesis, circadian rhythm, and sleep which could influence neurodevelopmental disorders (Saunders et al. 2023). In terms of intragenic localisation, the variants detected for *CACNA1D*,* CACNA1G* and *CNGB1* clustered within restricted regions of each gene. Specifically, the three *CACNA1D* (one novel and two ultra-rare) variants, cluster within exons 26–28, while all two *CNGB1* variants cluster within the cyclic nucleotide-monophosphate (c-NMP) binding domain (Fig. 1).

We did not detect variants in genes previously identified in GWAS, WES, or candidate gene association studies related to dyslexia, and none of the five genes highlighted in our study have been previously implicated in dyslexia. This confirms the highly polygenic nature of the condition. However, our annotations highlighted two unrelated individuals with distinct variants in *ZGRF1* (Supplementary Table 2), one of which was the same variant (4–112585555-C-T; c.G3913A; p.E1305K) previously reported in CAS, a language-related condition (Peter et al. 2016). The same 4–112585555-C-T variant was present also in a TD control and therefore studies of larger cohorts are required to clarify the role of this gene in language-related conditions.

The gene with highest number of recurrent variants observed in the discovery cohort was *CFTR*, which was not retained as a gene of interest because one of the variants detected in the discovery sample was also present in the TD group. Variants in *CFTR* are known to cause cystic fibrosis, and its role in neurodevelopment is not well established. However, early studies reported a significant linkage signal for language impairment at the *CFTR* locus (O’Brien et al. 2003).

These two examples highlight the importance of including controls with relevant phenotypic measures to highlight potential false positives. Conversely, the likelihood of false negatives was increased by the relatively small size of our sample, requiring stringent filtering criteria for pathogenicity predictions. Larger cohorts analysed with less stringent filtering criteria will enable a more systematic evaluation through formal statistical tests of the role or rare variants in dyslexia. The lack of formal case-control comparisons across our data is a limitation of our study. Furthermore, WGS approaches might also reveal effects outside the coding regions.

The detailed cognitive assessment of cases and their family members allowed us to further evaluate the phenotypic effects of the top five genes and variants. Although limited to four families, the segregation patterns were consistent with dominant effects for the three genes that were analysed. All variant carriers with available phenotypic data exhibited some degree of reading problems. In family 4, the *CNGB1* risk variant is transmitted from the father, who self-reported reading problems, to the proband but not to the sibling. The sibling presents with mild reading difficulties suggesting the presence of other risk factors. The patterns in family 10 and 18 are consistent with a dominant effect of the *CLDN3* 7–73769649-G-A variant on dyslexia and/or reading difficulties. In family 18, the variant is transmitted from the father with self-reported reading difficulties to three siblings with either dyslexia (PIQ z-score = 1.5; SWR z-score = −2.2; average reading measures z-score = −2.51) or mild reading problems. One of the siblings with a mild phenotype has a high PIQ (z-score = 1.7) and an average z-score across the reading measures of −0.54. The other sibling does not have a PIQ measure but presents with a high verbal IQ (z-score = 1.6) and an average z-score across the reading measures of −0.4. The fourth sibling does not have the variant and is a good reader (SWR z-score = 1.3). Finally, in family 32, the proband carries three risk variants and presents exceptionally low reading scores (average across six reading tasks = −2.5 SD) despite having a PIQ above the mean (z-score = 0.1). The sibling, who shares only the *CLDN3* variant out of the three, presents a mild phenotype characterised by a high PIQ (z-score = 2.3) and low z-scores on specific reading tasks (IWR = −0.61, ORTH = −1.16).

Large discrepancies between PIQ and reading scores were observed for the carrier of the *CLDN3* variant identified in the RD cases (PIQ z-score = 1.13; average reading z-scores = −2.23) and the carrier’s sibling (PIQ z-score = 1.6; average reading z-scores = −0.68) who shared the same variant. Overall, such patterns support a role for these rare variants in dyslexia and varying degrees of reading difficulties.

The phenotypic variability suggests that additional factors modulate the effects of these variants. It is worth nothing that, in the discovery cohort, the other two carriers of the *CLDN3* variant for whom we had no family data, also carried a high impact variant in either *CACNA1D* or *CACNA1G.* This suggests that the effect of the *CLDN3* variant on reading abilities could potentially be modulated by other rare risk variants. The phenomenon of variable expressivity, as well as incomplete penetrance, has been reported for most complex traits, including psychiatric and cognitive phenotypes (Kingdom and Wright 2022). These phenomena are likely to reflect the interaction of genetic, environmental, and lifestyle factors in influencing phenotypic outcomes. Typically, rare variants discovered in clinical cohorts with specific phenotypic characteristics tend to be associated with milder manifestations in the general population, likely due to the absence of such interacting risk factors. It is important to point out that, although the inclusion criteria for the present study were the same across the discovery and follow-up cohorts, these were derived from cohorts that were originally assembled via different recruitment criteria. Accordingly, the individuals in the discovery cohort presented on average more severe phenotypes compared to the follow-up cases (Table 1). Analysis in cohorts with phenotypic characteristics similar to the discovery cohort will provide more directly comparable datasets. However, to better understand genotype-phenotype relationships it will be crucial to analyse larger cohorts covering a broad range of the phenotypic distribution.

In conclusion, this study advances our understanding of potential roles of rare genetic variants in dyslexia. We identified five novel candidate genes. These include *CLDN3*, which plays several key functions in neurodevelopmental processes. Notably, a specific variant in *CLDN3* was identified in six independent cases. Two other genes, *CACNA1D* and *CACNA1G*, members of the VGCC gene-family, are involved in neuronal excitability, and have previously been linked to other neurodevelopmental conditions. While our findings do not overlap with previously reported dyslexia-associated genes, they align with the established role of VGCC genes in psychiatric and neurodevelopmental conditions. We extend the significance of this pathway to dyslexia, suggesting that VGCCs may contribute to its aetiology and linking these genes to shared mechanisms across neurodevelopmental traits. Additionally, genes such as *CFTR* and *CACNA1C*, which were excluded based on our stringent filtering criteria, may represent potential candidates for future investigations.

These findings highlight the importance of systematic WES or WGS analysis in larger, well-characterised cohorts to identify additional genetic factors and further advance our understanding of the neurobiology underlying dyslexia.

## Supplementary Information

Below is the link to the electronic supplementary material.

Supplementary Material 1

Supplementary Material 2

Supplementary Material 3

Supplementary Material 4

## Data Availability

All bioinformatic data produced in the present work are contained in the manuscript or in the supplementary material. The raw DNA sequencing data and individual phenotypic data are not openly available due to sensitivity reasons but are available from the corresponding author upon reasonable request. Code used in the current study to analyse the data can be found at [https://github.com/kmarianski/DyslexiaWES_CLDN3](https:/github.com/kmarianski/DyslexiaWES_CLDN3).
