# Supplementary material for: Whole-exome sequencing in children with dyslexia implicates rare variants in CLDN3 and ion channel genes
Source: Hum Genet. 2025 Dec 24;145(1):2. doi: 10.1007/s00439-025-02796-0 (PMC12738642; doi:10.1007/s00439-025-02796-0)
Supplement: Supplementary file 1 — Supplementary Material 1 [file 439_2025_2796_MOESM1_ESM.docx]

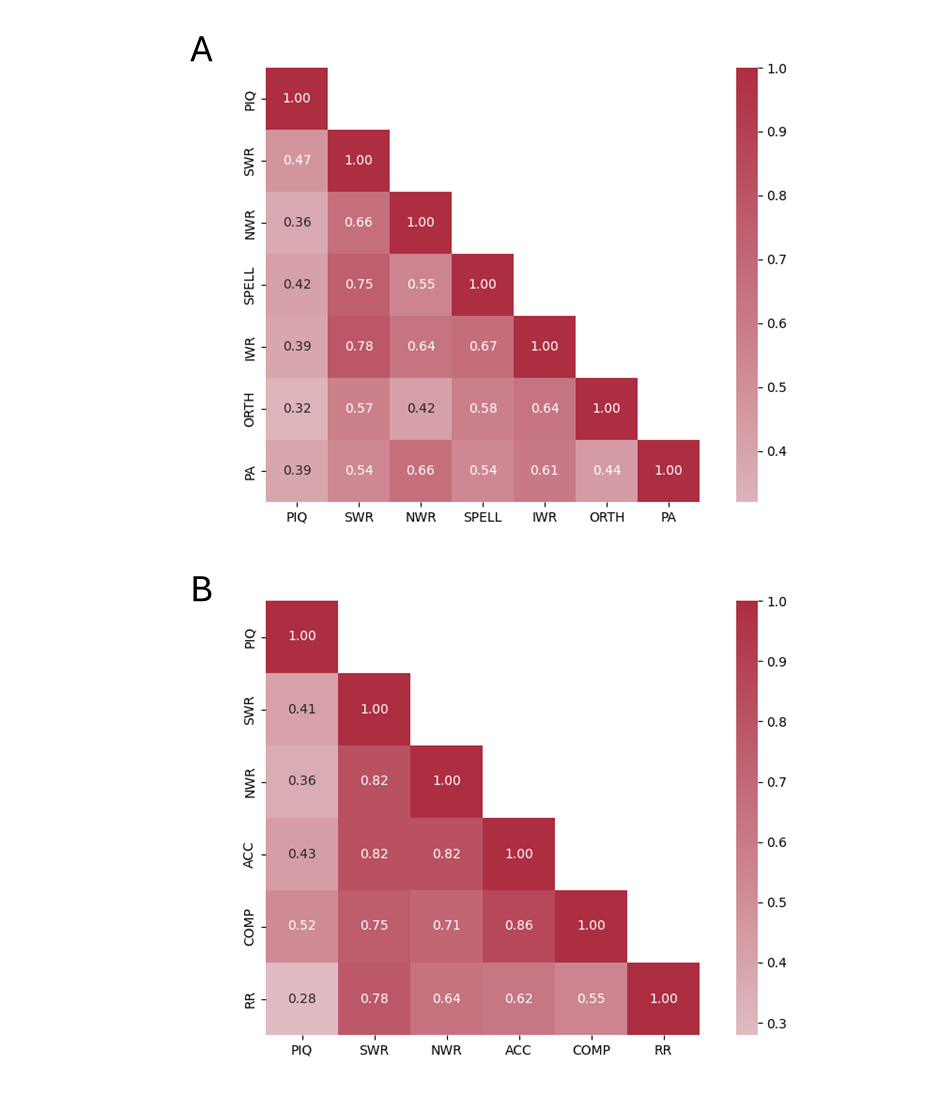


**Supplementary Figure 1.** Pearson correlation matrices for the reading measures calculated for A) the UK Dyslexia cohorts from which the discovery samples were derived and B) the UK Twin cohort from which the follow-up samples were derived.
