# Supplementary material for: Whole-exome sequencing in children with dyslexia implicates rare variants in CLDN3 and ion channel genes
Source: Hum Genet. 2025 Dec 24;145(1):2. doi: 10.1007/s00439-025-02796-0 (PMC12738642; doi:10.1007/s00439-025-02796-0)
Supplement: Supplementary file 2 — Supplementary Material 2 [file 439_2025_2796_MOESM2_ESM.docx]

**Supplementary Figure 3**. Visualisation of the expression of A) *CLDN3*, B) *CACNA1G*, C) *CACNA1D*, D) *CNGB1* and E) *CP* in single brain nuclei. The figures are screenshots taken from the Human Brain Protein Atlas database (https://www.proteinatlas.org/).

This resource contains brain cell type expression profiles based on single-nuclei RNA sequencing (snRNAseq) data covering 11 brain regions, including 2.5 million cells.
